# Preventing Depression: Investigation in Cohorts of its Etiology and Trajectory in Singapore - The PREDICT Study Protocol

**DOI:** 10.1192/j.eurpsy.2026.10579

**Published:** 2026-07-31

**Authors:** Y. Zhang, A. Jeyagurnathan, E. Samari, C. W. B. Tan, S. Gunasekaran, S. Archana, M. Ramu, S. Shahwan, R. Sambasivam, P. Satghare, J. A. Vaingankar, E. Abdin, N. Chandwani, Y. M. Mok, S. A. Chong, M. Subramaniam

**Affiliations:** 1 Research Division, Institute of Mental Health; 2 Saw Swee Hock School of Public Health, National University of Singapore; 3 Lee Kong Chian School of Medicine, Nanyang Technological University, Singapore, Singapore

## Abstract

**Introduction:**

Depression is a major contributor to global disability, with profound personal, social, and economic costs. In Singapore, while cross-sectional studies have provided valuable insights into the prevalence of depression, data on the long-term trajectory, outcomes, and determinants of depression remain scarce.

**Objectives:**

This protocol describes the PREDICT study, which seeks to address this gap by launching the nation’s first longitudinal cohort dedicated to major depressive disorder (MDD) and subsyndromal depression (SD). The objectives include examining disease prognosis, relapse, comorbidity, suicidality, treatment response, healthcare utilization, and mortality, identifying biopsychosocial determinants, quantifying economic burden, and understanding progression from SD to MDD.

**Methods:**

The study will recruit 3,201 participants aged 18–75 years, including those with first-episode depression, those with a longer history of depression, individuals with SD, and matched controls without psychiatric illness. Recruitment will be conducted at primary and tertiary care settings as well as from population cohorts. Participants will undergo comprehensive baseline and annual follow-up assessments for up to five years, capturing socio-demographic, psychosocial, physical, and biological data.

**Results:**

We hypothesize that sociocultural factors, such as accessible mental healthcare, social support, strong intergenerational relationships may confer protective effects leading to better outcomes compared with international cohorts, and that individuals with SD will incur higher resource use, poorer quality of life, and increased risk of progression to major depressive disorder as compared to controls.

**Conclusions:**

Findings will provide the first comprehensive longitudinal evidence on depression trajectories in Singapore. Results will guide clinical protocols, inform national mental health strategies (including Singapore’s Healthier SG initiative), and strengthen early detection and management of depression at the population level.

**Disclosure of Interest:**

None Declared

